# Effect of acidic polymers on the morphology of non-photochemical laser-induced nucleation of potassium bromide

**DOI:** 10.1038/s41598-024-58558-x

**Published:** 2024-04-05

**Authors:** Shuai Li, Xiongfei Xie, Yao Liu

**Affiliations:** Faculty of Materials Metallurgy and Chemistry, Jiangxi University of Science and Technology, Ganzhou, 341000 People’s Republic of China

**Keywords:** Non-photochemical laser-induced nucleation, Acidic polymer, Potassium bromide crystals, Nucleation probability, Crystal habit, Crystal number, Physical chemistry, Photochemistry, Optical physics

## Abstract

Non-photochemical laser-induced nucleation (NPLIN) in supersaturated potassium bromide (KBr) solutions with the addition of acidic polymers is reported here for the first time. Upon absorbing the incident laser, crystallites are immediately induced along the laser pathway in the solution, eventually growing into needle-shaped crystals of varying sizes. When comparing induction time, nucleation probability, and crystal habits with spontaneous nucleation, the results suggest that NPLIN creates a distinct morphological pathway, transforming cubic crystals into needle-like structures. Additionally, it improves crystallization probability and growth rate. This paper aims to realize control from crystal nucleation to crystal growth by adding acidic polymers to the process of laser-induced nucleation, potentially influencing crystal morphology modification in NPLIN. With 19 wt% acidic polymers added to the solution as additives, control over both crystal growth and morphological modifications was observed: cubic KBr crystals with square patterns were produced through laser irradiation, and there was a varying reduction in both the number and growth rate of the crystals. The influence of acidic polymers on the solution environment was analyzed to determine the reasons for the variations in crystal quantity and growth speed. The underlying mechanisms responsible for the changes in crystal shape were also discussed.

## Introduction

As an efficient method for refining and purifying chemicals, crystallization is widely employed in the production and preparation of various crystalline materials. Generally, crystallization is a heterogeneous process involving a solid–liquid interface and encompasses nucleation followed by crystal growth^1^. This process is marked by high theoretical complexity, and its underlying mechanisms are still poorly understood. The solid product obtained through crystallization requires a specific purity and crystal morphology, including size, shape, and polymorphism^2–4^. These characteristics determine the physicochemical properties of the product, such as density, hardness, melting point, solubility, dissolution rate, and bioavailability, as well as its processing, manufacturing, and economic value^5,6^. Therefore, obtaining crystals with the desired shape is crucial. Nucleation significantly affects the morphology, purity, and size distribution of crystals. Overcoming the stochastic nature of nucleation to selectively form nuclei is of great significance. Non-photochemical laser-induced nucleation (NPLIN) technology offers control over several aspects of nucleation: (1) it provides temporal control of nucleation through the energy and duration of the laser pulse; (2) the nucleation site can be controlled by changing the laser irradiation position and the laser beam pathway; (3) the number and size of crystals can be controlled by the number and energy of laser pulses; (4) it selectively induces the polymorphic form of crystals by changing the polarization of the laser^7^.

NPLIN was accidentally discovered by Garetz et al.^8^ during their work with urea, using a laser with a wavelength of 1064 nm. Since this wavelength falls in the nonabsorbing zone of the urea molecule, the photon energy generated by the laser pulse is too low to induce photochemistry. Its major advantage lies in its independence from photochemical reactions and the absence of chemical damage to solute molecules^9^. Alexander et al.^7^ provided a systematic overview of NPLIN by summarizing its discovery, putative mechanisms, and some observations of broader nucleation phenomena. NPLIN is typically used in aqueous solutions, including urea^10^, simple salts^11,12^, alkali halides^13,14^, glycine^15–18^, proteins^19,20^, and gaseous solutions like carbon dioxide in water^21,22^. Additionally, NPLIN has also been observed in pharmaceutical compounds dissolved in organic solvents^23,24^.

Three major mechanisms have been proposed in the literature to explain NPLIN: the Optical Kerr Effect (OKE)^25^, Dielectric Polarization (DP)^26^, and Nanoparticle Heating^21,22^. The Optical Kerr Effect suggests that solute molecules in disordered clusters become aligned due to the electric field generated by the laser pulse^25^. In the DP model, slightly subcritical solute clusters become polarized in the presence of an applied electric field, leading to stabilization and subsequent nucleation as they reach a critical state^26^. The Nanoparticle Heating mechanism posits that impurity particles, acting as mediums for crystal nucleation, absorb laser power. This absorption results in rapid heating around the impurity and the formation of nanoscale solvent vapor bubbles. These bubbles serve as sites for heterogeneous crystal nucleation of solute molecules, thus reducing the free-energy barrier and promoting nucleation^7^. However, these three proposed mechanisms can only partly explain the experimental observations reported. Given the complex and stochastic nature of nucleation, the mechanisms of NPLIN remain to be fully elucidated.

With the unprecedented control over nucleation that it offers, NPLIN technology has been utilized to explore crystal nucleation control^27^. Leveraging its ability to control crystalline polymorphism^28^, this technique has seen widespread development and application in the pharmaceutical industry^29,30^. The microfluidic device related to NPLIN, designed by Hua et al.^31^ enabled real-time and high-throughput in-situ characterization of crystal shape, size, and quantity on the chip. This device enhances material utilization and simplifies the research and analysis of crystal composition and crystallization kinetics. Korede et al.^32^ addresses the lack of large data sets on the NPLIN literature through a droplet-based microfluidic platform, supersaturation, wavelength, laser intensity, filtration and nanoparticles were investigated in potassium chloride aqueous solutions. NPLIN opens up new possibilities for controlling crystal nucleation and polymorphism. However, the crystallization process encompasses both nucleation and crystal growth. While NPLIN primarily offers control over crystal nucleation, the shape of crystals during the growth process can be designed using certain acidic polymers. This design is based on the theoretical principle that the growth rate of each crystal plane determines its specific morphology^33^. Therefore, control over the entire crystallization process becomes essential to obtain crystals with the desired shape. The combined use of NPLIN and acidic polymers was first applied by Liu and colleagues to investigate the crystal polymorphs of sodium acetate and cesium chloride^34,35^.

Numerous examples exist of using acidic polymers as additives to modify the growth and shape of inorganic crystals^36–40^. Moreover, the presence of these organic macromolecule additives significantly influences the role of NPLIN in morphological modification. In their work on the morphological control of sodium acetate crystals, Liu et al.^34^ utilized an integrated approach combining laser irradiation with acidic polymers. They found that specific nuclei could be easily induced by the laser, and the acidic polymers were effective in optimizing the crystalline morphology and size distribution. In another study by Liu et al.^35^ involving NPLIN, they successfully modified the morphology of crystals using this comprehensive technique. This approach allowed for complete control over nucleation and subsequent crystal growth. The work presented in this study aims to experimentally demonstrate how various aspects of potassium bromide (KBr) crystallization can be controlled. Variations of experimental results such as nucleation probability, growth rate, crystal morphology, and quantity are studied, influences of laser irradiation and acidic polymers on supersaturated KBr aqueous solution are analyzed for the first time. NPLIN can easily induce the initial crystallites in supersaturated KBr solutions, thereby providing control over nucleation. The addition of acidic polymers can influence solubility and crystal morphology, as well as modify crystal growth; combining this with laser-induced nucleation may result in unforeseen effects on crystal shape. Therefore, a comprehensive method that combines acidic polymers with NPLIN is employed to explore its effects on crystallization. This study experimentally investigates the impact of this comprehensive method on nucleation probability, crystal morphology, size, and quantity, and discusses the underlying mechanisms influencing crystal shape changes.

## Experimental methods

### Dilution of acidic polymer additives

In this study, polyacrylic acid (30 wt% aqueous solution, Macklin P816681-500 ml, LOT 1,531,430) (PAA) and polymaleic acid (50 wt% aqueous solution, Macklin P832909-500 g, LOT 1,531,430) (PMA) has been used as original organic additives. Another two additives with different mass fractions, 19 wt% PAA/PMA, were prepared by diluting the original additives in ultrapure water (18.2 MΩ cm). The solubility of potassium chloride in PAA and PMA can be negligible compared to its solubility in water. Thus, the water in added original additive and the quantity of ultrapure water were considered to be the entire solvent for KBr solute.

### Solutions and sample preparation

KBr (99%, Maclin P816700-500 g, LOT C14530307) and ultrapure water were used to prepare the samples, at a concentration of 0.73 g g^−1^, corresponding to a supersaturation of 1.07 at 25 °C. Concentrations are expressed as grams of solute per gram of solvent (g g^−1^). The solubility (C_sat_) of KBr solution at 25 °C is 0.68 g g^−1^. As solubility of KBr could be affected by acidic polymers, the value of solubility at 25 °C with 19 wt% PAA/PMA were tested, and the results indicated that the solubility of KBr increased with increasing mass fraction of acidic polymer, which are summarized in Table S1 and Fig. S1 of the Supporting Information. As solutions with a supersaturation of 1.07, which were initially tested, proved to be prone to nucleation through laser irradiation, the solutions were prepared with 19 wt% PAA or PMA in aqueous KBr solution, and the solubility was controlled to 1.07 (consistent with solution without additives). All solutions were placed in a temperature-controlled oven (70 °C) for 12 h to ensure complete dissolution. The heated solutions were then transferred into pre-cleaned glass sample vials (5 mL, Pyrex, 1.7 cm diameter, plastic screw-on caps with rubber inserts) by syringe, vials and syringes needed to be preheated. The vials were sealed and reheated for 2.5 h inside an oven (70 °C) to prevent any spontaneous nucleation events. The vials were placed in an incubator (25 °C) for 3 h, left untouched and slowly cooled to 25 °C before laser irradiation.

Three groups of KBr solutions in different mass fractions of additives are listed in Table 1, the experiment was divided into three groups, at least 70 identical samples were prepared for each group, half of the samples in each group remained unchanged in an incubator (25 °C) for 1 day to check spontaneous nucleation, another half were used for laser-induced nucleation experiments, and aging was unnecessary before laser irradiation, the number of nucleated samples were counted. Group 1 was the original solution without additives, using for reference; the additives was PAA (19 wt%) in the Group 2 and PMA (19 wt%) in Group 3, respectively.

**Table 1:** Three groups of KBr solutions in different mass fractions of additives.

Group	Additive	Actual mass fraction
1	None	0–no additives added
2	Polyacrylic acid (PAA)	19 wt%
3	Polymaleic acid (PMA)	19 wt%

### Laser-induced nucleation

Sample vials stored at the incubator of 25 °C for 3 h were then placed in the laser pathway, the laser pulse (10 Hz, 6 ns duration) with 532 nm wavelength was generated from a 1064 nm Q-switched Nd^3+^: YAG laser (Quantel Q-smart 450) with a harmonic generator (2ω) for frequency doubling. The laser beam was then passed through a variable beam reducer to shrink the beam diameter from 2.5 to 1.0 mm. The mean power of the unfocused beam was measured using a power meter (Ophir, Nova II) to adjust laser power. In addition, as laser passed through a cylindrical glass sample bottle, the beam was focused by a cylindrical lens. The maximum time of laser irradiation was 1 s, the laser pulse would be immediately stopped when as soon as the crystals were observed. The laser power was 1.0 mJ pulse^−1^ with 60.7 MW cm^−2^ energy density. The details of experimental setup can be found in Fig. S2 of the Supporting Information.

### Crystals characterization

KBr crystals produced from different experimental groups were identified by X-ray powder diffraction analysis (Rigaku Miniflex 600). To obtain their X-ray diffraction (XRD) patterns, some crystals with representative morphology were taken out of the crystallization vials and the samples were prepared according to the following steps: crystals were filtered out and dried after 4 h of growth, then these crystals were ground into powder and sealed into small sample bags before XRD analysis. Spontaneous crystals, laser-induced crystals with and without acidic polymers, were identified using X-ray powder diffraction analysis (20 mA, 40 kV, 20 ~ 25 °C). Data were collected from 10 to 90° 2θ in steps of 0.0204°, and a scan rate of 0.004°/s.

## Results

### Spontaneous nucleation and NPLIN

Half of the samples in Group 1 were used to check spontaneous nucleation probability and another half were used for NPLIN, the nucleation probabilities are summarized in Table 2. 16 out of 35 samples were spontaneous nucleated, with the probability of 45.7%; 34 out of 35 samples were crystallized through NPLIN, with the nucleation probability of 97.1%.

**Table 2:** Number and probability of nucleation for Group 1.

	Number of nucleated samples	Nucleation probability (%)
Spontaneous	16	45.7
NPLIN	34	97.1

In systems without acidic polymers, spontaneous nucleation requires at least 2 h of induction time, whereas laser-induced nucleation takes less than 1 s (10 pulses).

**Figure 1:**
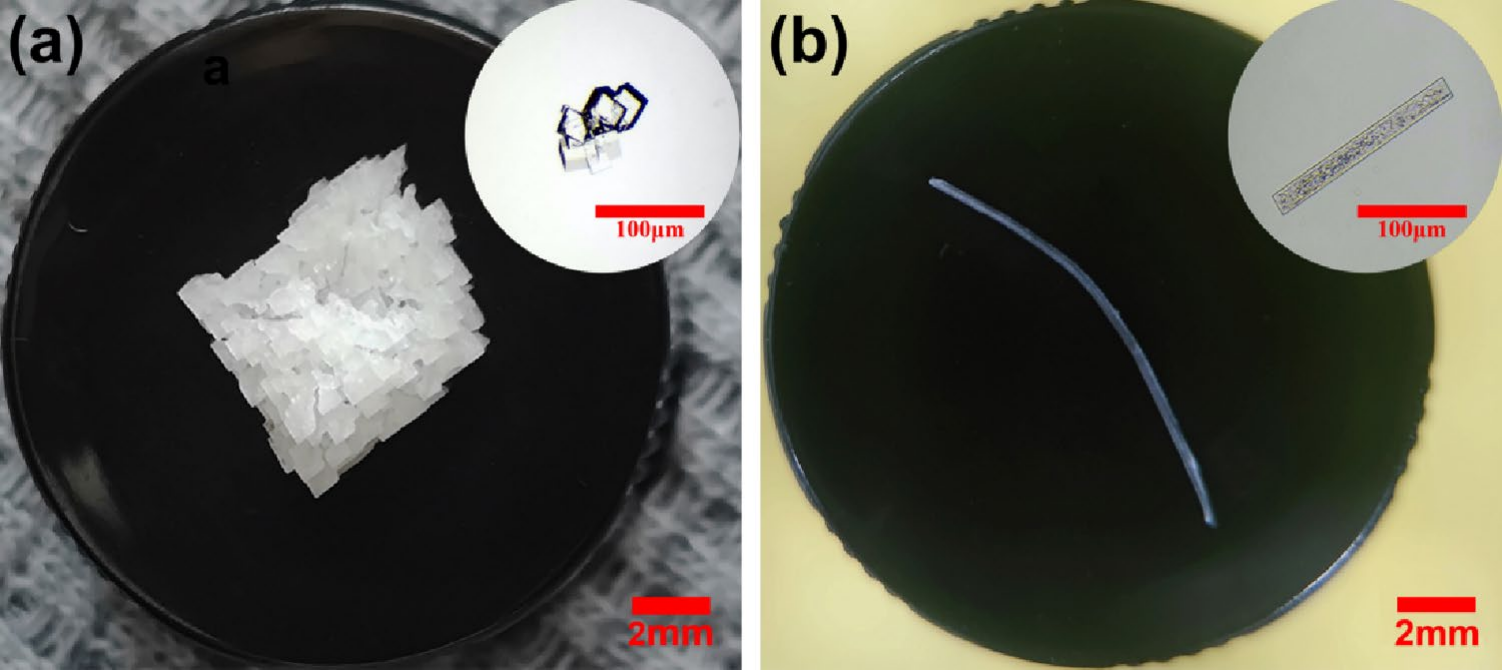
Images of spontaneous and laser-induced crystals from solutions without additives. (**a**) cubic crystals obtained from spontaneous nucleation without additives; (**b**) needle-shaped crystals induced from laser without additives by laser. The inset photo in the upper right corner of (**a**) and (**b**) are spontaneous and laser-induced initial crystallites, respectively.

The morphology of KBr crystals was observed through optical microscope. As can be seen in Fig. 1, all crystals achieved from spontaneous nucleation were cubic with lengths around 0.5–1.0 mm, these cubic crystals easily coalesce to form irregular large polycrystal blocks; whilst 100% of the laser-induced KBr crystals were needle-like crystals with lengths around 0.6–1.4 cm, indicating that the crystalline morphology could be transferred through laser irradiation. The growth process of needle-like KBr crystals along the laser pathway can be classified into three steps: (1) nuclei appear along the laser pathway; (2) KBr solute molecules gather around the initial crystal and the crystal grows rapidly; (3) the crystals settle to the bottom and eventually form crystals with needle-like structure. It’s noteworthy that each needle-like crystals exist independently of each other and does not agglomerate with others, grows rapidly and stops growing within 20 min after laser irradiation, these steps in schematic illustration are detailed the Supporting Information (Fig. S3).

### Effect of acidic polymers on crystallization

Compared to the nucleation probability in Group 1 (without acidic polymers), the probabilities of both spontaneous and laser-induced nucleation significantly declined in Groups 2 and 3, which contain the acidic polymers PAA and PMA, as seen in Table 3. Specifically, spontaneous nucleation probability was reduced from 45.7% in Group 1 to 14.3% and 22.9% in Group 2 and Group 3, respectively; the probability of laser-induced nucleation was reduced from 97.1% in Group 1 to 57.1% and 77.1% in Group 2 and Group 3, respectively. Through these comparisons, the nucleation probability was significantly reduced under the effect of PAA or PMA, indicating that the crystallization was inhibited to some extent by acidic polymers in solutions. As shown in Table 3, the probability sequence of spontaneous or laser-induced nucleation of the three solutions is Group 2 (PAA) < Group 3 (PMA) < Group 1 (Aqueous).

**Table 3:** The probability of spontaneous or laser-induced nucleation of Group 2 and Group 3.

Group	Method	Nucleation number	Nucleation probability (%)
2 (19 wt% PAA)	Spontaneous	5	14.3
	NPLIN	20	57.1
3 (19 wt% PMA)	Spontaneous	8	22.9
	NPLIN	27	77.1

In Groups 2 and 3, spontaneous nucleation usually took more than 4 h, whereas laser-induced crystals could be observed after 3 s (30 pulses) of laser irradiation.

**Figure 2:**
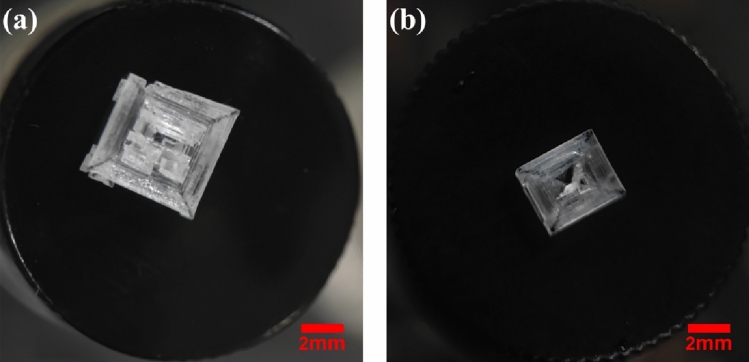
Images of spontaneous and laser-induced crystals from solutions 19wt% PAA. (**a**) crystals obtained from spontaneous; (**b**) cubic crystal with square patterns induced by laser.

With the effect of acidic polymers, another morphology of KBr crystals were observed for the first time. The results of crystals achieved by spontaneous or NPLIN from Group 2 (PAA) were summarized in Fig. 2, these cubic crystals do not cluster during crystal growth. With the presence of 19 wt% PAA, the crystallites of Group 2 were larger and more rigid than those of Group 1, the final crystals were cubic with larger lengths around 2.11–6.07 mm, indicating that the growth pathway of crystals could be modified through acidic polymer.

In addition, the presence of acidic polymers leads to different nucleation probabilities. Crystals in samples without additives can be induced by several pulses, the crystal growth takes around 20 min for the whole solution to form a crystalline structure, whilst first crystal in the solution with PAA was observed at least ten minutes after laser irradiation, the duration of complete crystal growth takes around 3 h, the result revealed that the crystal growth rate was significantly reduced by the addition of PAA. Compared to the same mass fraction of PAA, PMA has the similar effect on nucleation probability and crystal morphology, while the duration of crystal growth was about 2 h. The crystals induced by laser in Group 3 (PMA) were large cubic crystals, but these crystals are not rigid enough as some crystal fragments easily fall off from the crystals, leading to the resulting crystals being incomplete, the images of these crystals are given in Fig. S4 of the Supporting Information. From these results, the change on crystal growth rate, nucleation probability, and morphology is related to acidic polymers, the inhibition of PAA on nucleation probability and crystal growth rate was more obvious than PMA when their mass fractions are the same.

## Discussion

The results show that acidic polymers could provide several ways to control crystal growth parameters, including nucleation probability, crystal growth rate and crystalline morphology after NPLIN. In general, nucleation probability decreases with the increase of the mass fraction of additives; crystal growth rate is inversely proportional to the concentration of acidic polymers; cubic crystals and needle-shaped crystals can be induced by lasers with and without PAA/PMA, respectively. Here, the effects of acidic polymers on nucleation probability, crystal growth rate, and crystal number are analyzed and inferred, and the underlying mechanisms of crystal morphology change are explained.

### Effect of acidic polymers on nucleation probability and growth rate

As shown in Tables 2 and 3, the nucleation probability with PAA/PMA was largely lower compared to the solution without acidic polymers, these findings were consistent to the research done by Liu et al.^34,35^ several acidic polymers with different mass fractions were used to uniformly control the morphology, number and size of crystals induced by laser. The nucleation event in this study using the combination of NPLIN and acidic polymers still could be explained by the free energy change *ΔG (r, E)*^7,11,41^, the function *ΔG (r, E)* is given by.

1 $$\Delta G(r, E )=4\pi {r}^{2}\gamma -\frac{4}{3}\pi {r}^{3}\left(A\mathit{ln}S+a{E}^{2}\right)$$

Here, *r* is the radius of a subcritical cluster, γ is the solution—crystal interfacial tension, $A={\rho }_{s}RT/M$, where ${\rho }_{s}$ is the density of the of the crystal, *R* is the universal gas constant, *M* is the molar mass of the crystal and *T* is the temperature, *E* is the electric field induced by the laser, and *a* is decided by the dielectric constants of the particle $({ \varepsilon }_{s})$ and solvent $({ \varepsilon }_{L})$, the function of $a{E}^{2}$ is given by.

2 $$a{E}^{2}=\frac{3{j}_{peak}}{c}\left(\frac{{{\varepsilon }_{S}-\varepsilon }_{L}}{{{\varepsilon }_{S}+\varepsilon }_{L}}\right)$$

Where *j*_*peak*_ is the peak power density of the laser, the parameter c the speed of light in vacuum (3 × 10^9^ m s^−1^). From Eqs. (1) and (2), the critical radius ${r}_{c}$ of a cluster becoming a nucleus is written by.

3 $${r}_{c}(E)=\frac{2\gamma }{A{\text{ln}}S+a{E}^{2}}$$

As the value of *c* is much higher than the other parameters in Eq. (2), the value of $a{E}^{2}$ has a negligible effect on the critical radius, thus, the value of ${r}_{c}$ can be approximated as.

4 $${r}_{c}=\frac{2\gamma }{A{\text{ln}}S}$$

In systems with acidic polymers, the interfacial tension between the additive and the crystals has to be considered in addition to the interfacial tension between the solvent and the crystals, in the presence of acidic polymer, the interfacial tension (*γ’*) between the solution and crystal is increased^34,35^. In addition, functional groups from acid polymer may adsorb on the nuclei or crystal surfaces of KBr, which may be a key factor leading to increased interfacial tension ^34,42^, correspondingly increasing the value of free energy change (*ΔG’*) and critical radius (*r*_*c*_*’*) with respect to Eqs. (1) and (4), this upward trend in *ΔG’* in the presence of acidic polymers disfavors the formation of crystal nucleation, leading to a decrease in the nucleation probability. Thus, the addition of acidic polymers could decrease nucleation probability, which is consistent to the result shown in Tables 2 and 3.

The period of crystal growth with acidic polymers was extended to 2 h compared to 20 min in the absence of additives, crystal growth rate was largely declined after the addition of acidic polymers. This tendency is in agreement with the study of sodium acetate^34^, acidic polymers are macromolecular substances which could increase viscosity, similarly, viscosity can also be increased by the addition of basic and neutral polymers. When the viscosity increases, molecular mobility in solution can be significantly slowed down, which could hinder the diffusion process of solute molecules, corresponding reducing the crystal growth rate. In a work involving polyacrylate modified sodium oxalate crystallization, Fu et al.^42^ pointed out that polymer anions could bind into the crystalline matrix, this implied that the functional group could capture a small fraction of the K^+^ ions, indicating that the low crystal growth rate could be caused by PAA/PMA.

**Figure 3:**
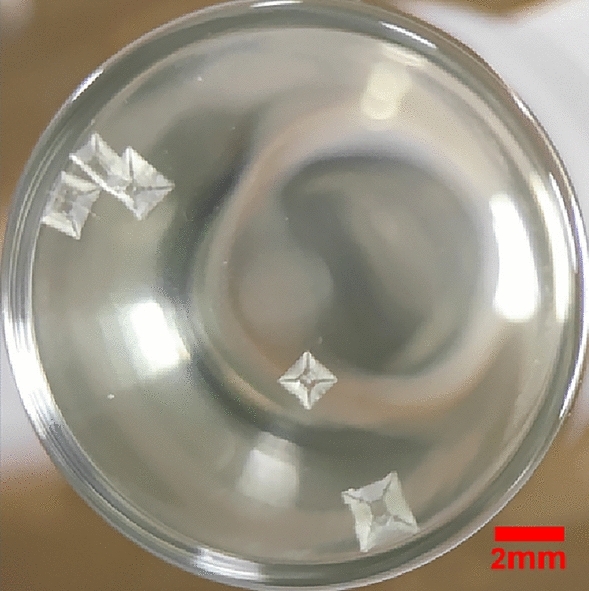
Crystals in the bottom view of vials induced by laser with the effect of 19 wt% PAA.

Compared with crystals in Fig. 1a, the number of crystals induced by laser could be substantially diminished by acidic polymers (Fig. 3); Compared the number of crystals in PAA with 19–10wt% (Section S5 of Supporting Information), it shows that the lesser mass fraction of acidic polymers, the higher quantity of crystals could be obtained, the mass fraction of acidic polymers could specifically control the number of crystals. As *r*_*c*_*’* increases after the addition of acidic polymers, more solute molecules are required to form critical clusters, indicating that the solutions with PAA/PMA could contain less quantities of nucleation sites to form critical clusters. Thus, only fewer crystals could be formed after laser irradiation with a high mass fraction of acidic polymers, this is aligned to the result in the work of cesium chloride^35^, acidic polymers were observed that could significantly decrease nucleation sites, leading to a decrease in the number of crystals induced by laser.

### Effect of acidic polymers on crystal morphology

**Figure 4:**
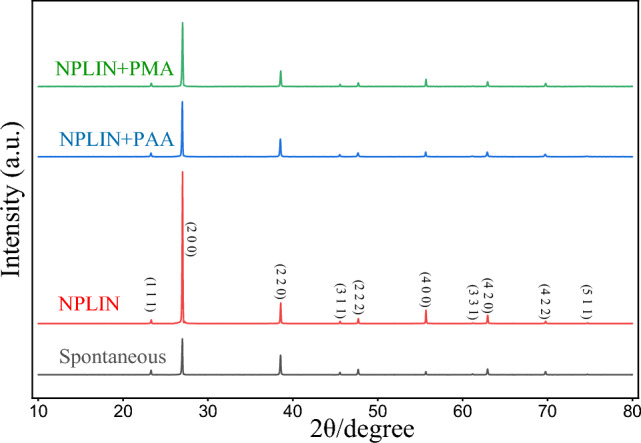
Powder X-ray diffraction patterns with crystals induced by four methods are marked on the graph. The sample with gray curve was induced by spontaneous; the sample with red curve was induced by NPLIN; the sample with blue curve was induced by NPLIN and PAA; the sample with green curve was induced by NPLIN and PMA.

The X-ray powder diffraction patterns of the crystals are shown in Fig. 4, including spontaneous crystals, laser-induced crystals without additive and with PAA/PMA. According to the observation results of XRD diffraction patterns, the characteristic powder diffraction peaks in each XRD pattern are approximately the same, which indicates that the main component of the samples are KBr and no new polycrystalline forms are produced. In addition, the intensity of each characteristic peak was slightly different, as the morphological changes of the four crystals depend on laser irradiation and acidic polymer, it can be hypothesized that the acidic polymer and NPLIN could modify the crystal shape. As shown in Fig. 4, compared to spontaneous crystals, the intensity of the main peak 27° (200) of the laser-induced crystals is significantly higher, suggesting that the growth rate of this crystal plane is promoted by laser energy. Based on the intensity of character peak and crystal shape changes, it can be inferred that lasers and acidic polymer provide morphological control through affecting the growth speed of specific crystal plane.

**Figure 5:**
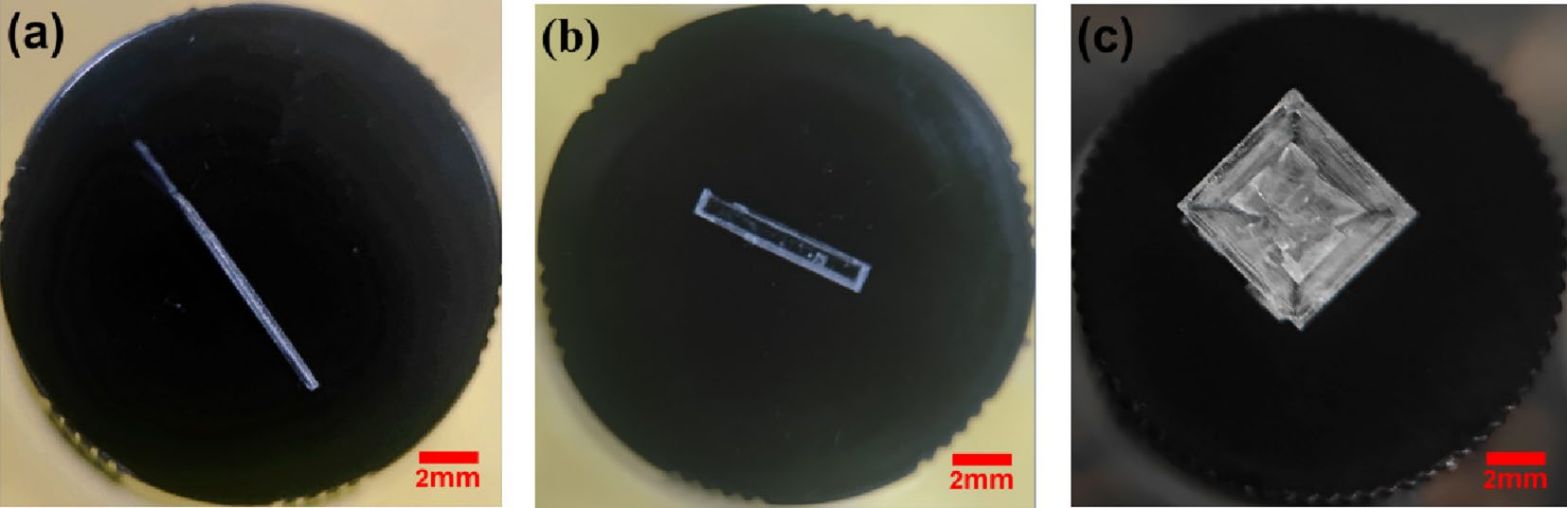
Three crystal images. (**a**): needle-shaped, NPLIN without PAA; (**b**): bar-shaped, NPLIN with 10 wt% PAA; (**c**): cubic, NPLIN with 19 wt% PAA. The laser-induced crystal shape depends on the mass fraction of the acid polymer.

As shown in Fig. 5a, needle-shaped crystals are achieved by laser irradiation without acidic polymers. It’s different from spontaneous nucleation, NPLIN induced multiple crystal nuclei in a row along the laser beam pathway, these single crystals grow individually to form crystals with a long shape. In addition, the difference from the cubic shape in Fig. 5c may be attributed the lower viscosity of the system. Due to the relatively low viscosity of the original solution, the solute molecules have a tendency to initially assemble at the edge and point of crystal^35^. Compared to the addition of acidic polymers, the number of solute molecules for each crystal nucleus to form a complete crystal is insufficient as more nucleation sites are induced by laser and the supersaturation is fixed, then the solute molecules would only aggregate on the edge or point of crystal, resulting in the formation of needle-liked crystals. As acidic polymers have upward tendency on several aspects including viscosity, interfacial tension, and critical radius^34,35^. In the presence of 19 wt% PAA, crystal growth is complete due to fewer nucleation sites induced by laser and the sufficient number of solute molecules to form complete crystals, this is why cubic crystals are formed, shown in Fig. 5c. Whilst at lower mass fraction of PAA (10 wt%), both cubic and bar-shaped crystals (Fig. 5b) were observed after laser irradiation, bar-shaped crystals are similar to the needle-like crystals, this is due to the reason that the number of nucleation sites is between in original solution and that in 19 wt% PAA, which is detained in Section S5 in supporting information.

These morphology change also could explained by Gibbs and Wulff et al.^43^ the research showed that the crystal will adjust its shape when approaching equilibrium in order to minimize its own total surface energy to a minimum, in other words, at constant temperature and pressure equilibrium, a certain volume of crystal has the minimum total surface energy, and their theory can be described by the following equation:

5 $$\Delta {G}_{s \,min}=\int \gamma \,(i) \,dA$$

where *ΔG*_*s min*_ is the total surface energy of the crystal in equilibrium state; A is the surface area of the crystal; *γ(i)* is the interfacial energy, it is defined as the total free energy per unit area of the interface, and it is numerically equivalent to the interface tension, interface tension is defined as the work required to form a new interface over the unit area of the solution, thus, the total surface energy of the crystal is the calculus of interfacial energy and area.

As the increase in viscosity and interfacial tension of the solution with acidic polymers, according to the Eq. (6), the total surface energy would increase with the increase of interfacial energy, which matches the experimental trend, the relative surface area order of the three crystals is cubic > bar-shaped > needle-shaped, in contrast to the ordering of their additive concentrations.

In addition, one of the mechanisms by which acidic polymers causing crystal shape modification is adsorption on a specific face of the crystals to inhibit crystal face growth^33^. In generally, without additives, spontaneous KBr crystals are small cubic crystals, bar-shaped and needle-shaped crystals might be obtained by suppressing the different crystal planes of the cubic crystals.

## Conclusion

In summary, the effect of acidic polymers on NPLIN in supersaturated KBr solutions was investigated, focusing on several aspects including nucleation probability, crystal growth speed, size, and morphology. The results indicated that NPLIN enhances the crystallization rate compared to the spontaneous approach by reducing the induction times and growth period. Crystallites were observed along the laser pathway immediately after laser irradiation, and they fully grew within 20 min. Regarding crystal morphology, spontaneous nucleation typically results in aggregates of cubic crystals, whereas laser-induced nucleation produces needle-shaped crystals that exist independently of each other. Additionally, acidic polymers have significant effects on crystal morphology and growth in conjunction with NPLIN. The presence of PAA extended the crystal growth time to 3 h, reduced the number of crystals to a few grains, and led to the production of large and strong cubic crystals. The decreased crystal growth speed could be attributed to the increased viscosity of the solutions caused by acidic polymers. This increase in viscosity raises the interfacial tension between the solution and crystals, which in turn increases the radius of a subcritical cluster, leading to a reduction in the number of crystals. The differential growth speeds of various crystal planes, caused by the adsorption of organic macromolecules on specific planes, alter the crystal shape. An increase in interfacial energy, in a bid to maintain a constant total surface energy, may lead to self-regulation of crystal shape and the creation of new crystal morphologies.

### Supplementary Information

Supplementary Information.

## Data Availability

All data generated during this study are included in this article and its supplementary information files.
