# Supplementary material for: Effect of acidic polymers on the morphology of non-photochemical laser-induced nucleation of potassium bromide
Source: Sci Rep. 2024 Apr 5;14:8051. doi: 10.1038/s41598-024-58558-x (PMC10997761; doi:10.1038/s41598-024-58558-x)
Supplement: Supplementary file 1 — Supplementary Information. [file 41598_2024_58558_MOESM1_ESM.docx]

**Effect of Acidic Polymers on the Morphology of Non-Photochemical Laser-Induced Nucleation of Potassium Bromide**

**Shuai Li,^a^ Xiongfei Xie,^a^ and Yao Liu^a,^ ***

^a^Faculty of Materials Metallurgy and Chemistry, Jiangxi University of Science and Technology, Ganzhou 341000, People’s Republic of China.

*Corresponding author. E-mail: [yao.liu@jxust.edu.cn](mailto:yao.liu@jxust.edu.cn). *ORCID: 0000-0003-2402-7841

1. **Solubility and spontaneous nucleation of KBr in different solvents:** It is observed that the solubility of KBr is almost independent from acidic polymers, its solubility in water is slightly altered by additives. The solubility of KBr in water with or without acidic polymers (19 wt% PAA/PMA) was determined, especially under experimental conditions (25 ℃). The experimental solubility is summarized in Table S1 and Fig. S1. The unit of solubility is expressed as grams of solute per 100 grams of solvent. To demonstrate the difference in morphology between laser-induced nucleation and spontaneous nucleation, a specific supersaturation must be selected for convenient study and observation of spontaneous nucleation. A series of supersaturated solutions (S = 1.00 - 1.10) were cooled to 25 ℃ and left untouched for 24 hours to observe if spontaneous nucleation occurs, the results showed that at the solutions with 19 wt% acidic polymers are prone to spontaneously crystallize at 25 ℃ with supersaturation higher than 1.05 (water) and 1.06 (19 wt% PAA or PMA).

**Table S1.** KBr solubility at different solvents and temperatures.

|  | KBr solubility (g 100g^-1^) | | | | |
| --- | --- | --- | --- | --- | --- |
|  | 5℃ | 15℃ | 25℃ | 35℃ | 45℃ |
| Water | 56.5 | 62.5 | 67.8 | 69.1 | 74.5 |
| 19 wt% PAA | 58.0 | 64.5 | 69.1 | 72.3 | 74.9 |
| 19 wt% PMA | 59.0 | 65.0 | 69.7 | 72.7 | 75.2 |


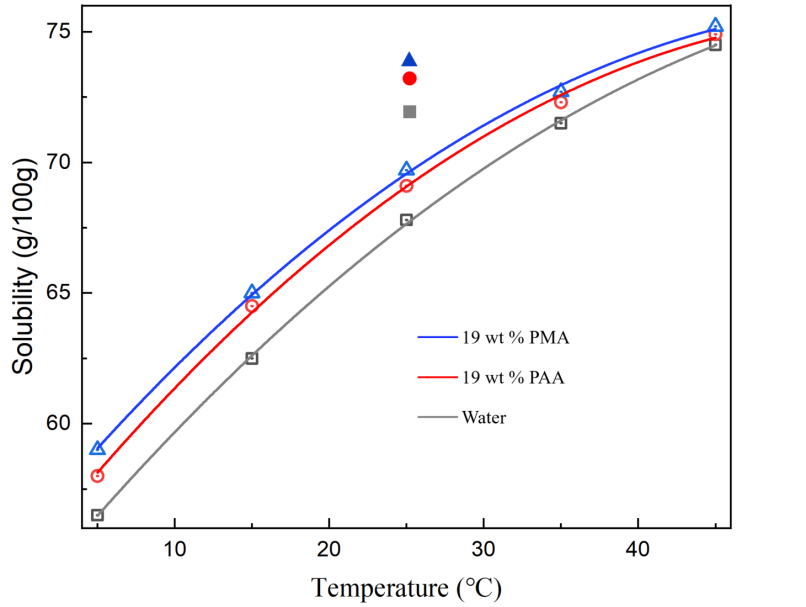


**Figure S1**. Schematic illustration of solubility of KBr. Solubility in water (gray curve); Solubility with 19 wt% PAA (red curve); solubility with 19 wt% PMA (blue curve). The solid points represent the concentrations of spontaneous nucleation at 25 ℃ with aqueous solution (gray), 19 wt% PAA (red) and 19 wt% PMA (blue).

1. **Schematic layout of the optical setup.** The experimental setup is shown in Fig. S2. The laser light was generated by a Q-switched Nd^3+^: YAG laser (Quantel Q-smart 450) with a self-contained harmonic generator (2ω) for frequency doubling. The power of the laser beam is controlled by a beam attenuator module (BAM). Beam pathway is regulated by two coated laser mirrors. Before the laser passes through the sample, a variable beam reducer is used to reduce the beam diameter to 1.0 mm. After measuring the average laser power with a power meter (Ophir, Nova II), the power is adjusted to the appropriate range.


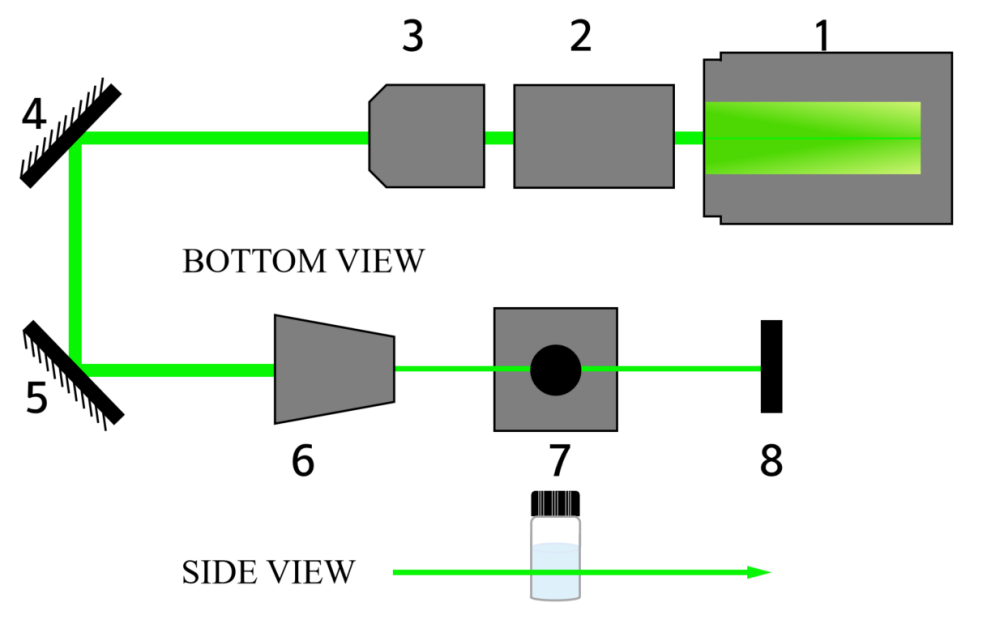


**Figure S2**. Schematic representation of the experimental setup for laser irradiation of KBr: (1) Nd^3+^: YAG laser source; (2) beam attenuator module; (3) harmonic generator; (4, 5) coated laser mirrors; (6) variable beam reducer; (7) the vials placed on a sample holder; (8) power meter.

1. **Schematic illustration of crystal growth after laser irradiation.** With the effect of laser irradiation, in a supersaturated (S=1.07) KBr solution without additives, the crystals grown in laser pathway are needle-like, as summarized in Fig. S3. The crystal growth process can be divided into three steps: the initial occurrence of crystal nucleus along the laser pathway (step a); solute molecules assemble at the edge of initial crystal (step b) and the crystal grows rapidly, the crystals settle to the bottom and continues to grow, forming needle-like crystals after complete growth (step c).


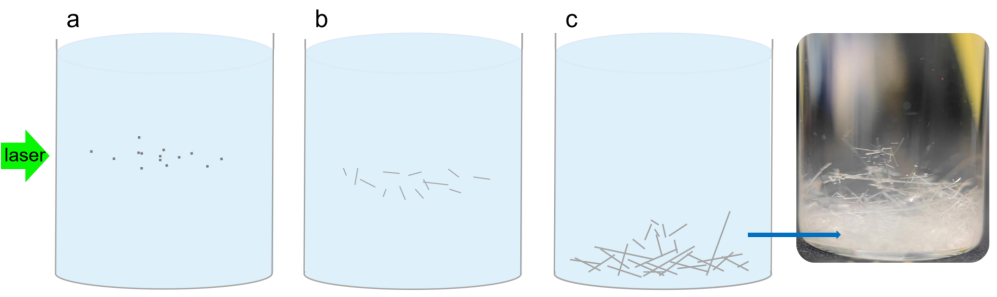


**Figure S3.** Schematic illustration of crystal growth pathways in samples without additive after laser pulse irradiation. The pathway contains three steps of crystal growth: a large number of crystal nuclei are induced by laser (a); then they gradually grow into needle-shaped crystals (b); The needle-shaped crystals become thicker and longer at the bottom over time (c).

1. **Images of crystals in solution with PMA.** Group 3 solution contained 19 wt% PMA, with the influence of additive, KBr crystals induced by laser irradiation were cubic-shaped, as is shown in Fig. S4a. The crystals from the bottom of vials are cubic but damaged and fragile (see Fig. S4b).


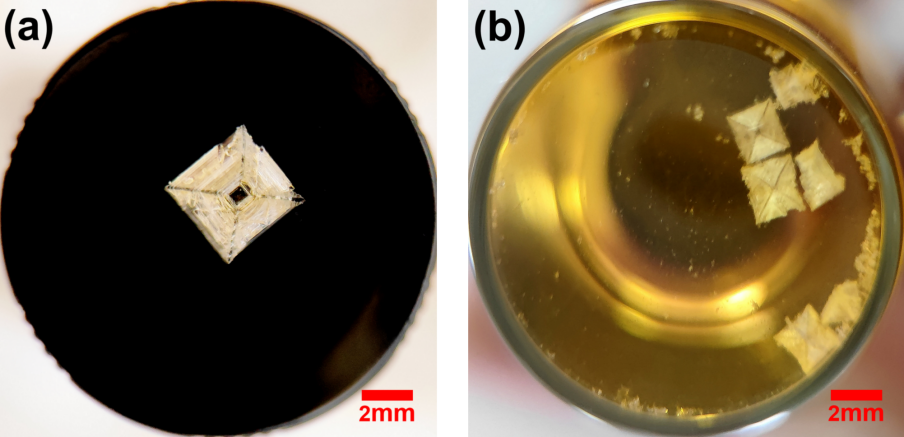


**Figure S4.** Images of crystal induced from solutions with 19wt% PMA through NPLIN.

1. **Crystals obtained through laser irradiation from 10 wt% PAA.** Both bar-shaped and cubic crystals were achieved from 10 wt% PAA (Fig. S5) similar to the morphology of KBr crystals in 19 wt% PAA, whilst the number of crystals (at least 50) in 10 wt% PAA were higher than that in 19 wt% PAA. The number of crystals obviously decreased with the increase of PAA mass fraction, which revealed that acidic polymers can inhibit the nucleation probability from laser irradiation.


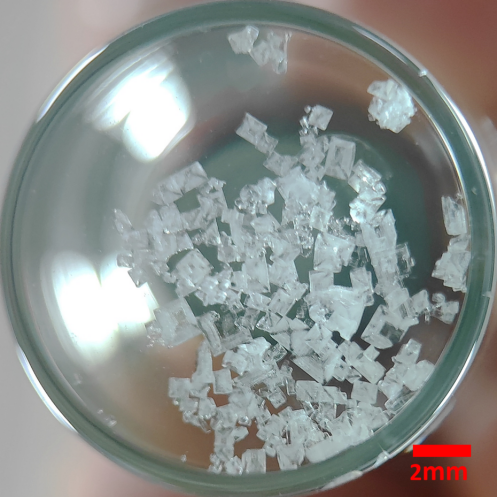


**Figure S5.** The bottom image of crystals achieved from laser with the effect of 10 wt % PAA.
